# Isotemporal Substitution Associations of 24 h Movement Behaviors and Creative Thinking in School-Aged Children: A Compositional Data Analysis

**DOI:** 10.3390/bs16101873

**Published:** 2026-10-11

**Authors:** Zhengmao Guo, Lingxiao Li, Zhihui Li, Ming Wu, Jian Yang

**Affiliations:** 1 School of Physical Education, Shanghai Normal University, Shanghai 200234, China; zmguo@shnu.edu.cn (Z.G.); 1000589339@smail.shnu.edu.cn (L.L.); 2 School of Physical Education, Soochow University, Suzhou 215001, China; 3 School of Kinesiology and Physical Education, Zhengzhou University, Zhengzhou 450001, China; wumingzzu@126.com; 4 College of Physical Education and Health, East China Normal University, Shanghai 200241, China; yangjianzx@sina.com

**Keywords:** physical activity, sedentary behavior, compositional data analysis, isotemporal substitution, creative thinking

## Abstract

This cross-sectional study examined associations between 24 h movement behavior composition and creative thinking in school-aged children, using accelerometers, the Torrance Tests of Creative Thinking, compositional linear regression, and compositional isotemporal substitution analysis. A total of 319 school-aged children (168 boys, 151 girls; mean age = 10.06 ± 1.37 years; Grades 1–5) from Shanghai, China, were included. Relative time in moderate-to-vigorous physical activity (MVPA; β = 16.019, 95%CI [9.472, 22.578], *p* < 0.001) and sleep (SLP; β = 6.374, 95%CI [3.263, 9.492], *p* < 0.001) was significantly positively associated with total creative thinking scores, whereas sedentary behavior (SED; β = −11.621, 95%CI [−19.345, −3.906], *p* < 0.01) was significantly negatively associated; light physical activity (LPA; β = −4.284, 95%CI [−8.771, 0.207], *p* = 0.062) was negatively but nonsignificantly associated. Reallocating time from LPA or SED to MVPA or SLP was associated with significant increases in creative thinking, and reverse reallocations with significant decreases. These associations were asymmetric: scores rose gradually with the former reallocations but fell rapidly with the latter. For both MVPA and SLP, replacing SED yielded greater model-estimated gains than replacing LPA. These findings suggest that promoting creative thinking may benefit from an integrated approach to 24 h movement behaviors: increasing MVPA, ensuring sufficient SLP, reducing SED, and managing LPA. Time may be reallocated from SED and LPA to MVPA and SLP, while guarding against reverse reallocations. Future longitudinal and intervention studies are needed to determine whether reallocating time causally changes creative thinking.

## 1. Introduction

As a higher-order form of human intellectual activity, creative thinking is a central driving force behind scientific progress, technological innovation, and social change. In the era of the knowledge economy, cultivating creative thinking has become a key issue in national talent development strategies. The school-age period (6–12 years) is a critical window for children’s cognitive development. Creative thinking during this period differs both from the lower-order form in preschool children, which is characterized primarily by intuitive imagination, and from the higher-order form in adults, which is supported by rigorous logic and systematic methods; it has its own mechanisms of emergence and patterns of development. However, existing research on children’s creative thinking has largely focused on teaching strategies and curriculum design in school settings (Samaniego et al., 2024; Lin, 2024; C. Wang & Sun, 2018; Gregory et al., 2013), with little attention to behavioral factors within the structure of children’s daily lives that influence the development of creative thinking. In fact, children’s 24 h day is occupied by behaviors such as sleep (SLP), sedentary behavior (SED), light physical activity (LPA), and moderate-to-vigorous physical activity (MVPA). The allocation of time to these behaviors and the ways in which they are combined constitute a finite, interdependent ‘behavioral ecosystem’. How to optimize the configuration of behaviors within this system to promote the development of creative thinking in school-aged children is a theoretical and practical issue that urgently needs to be addressed at the intersection of developmental psychology, exercise science, and education.

In recent years, 24 h movement behaviors have received widespread scholarly attention as an integrated unit of analysis. The World Health Organization and guidelines from several countries have explicitly recommended that school-aged children engage in at least 60 min of MVPA daily, limit SED, particularly screen time, and obtain sufficient SLP (Wei & Chen, 2024; J. Wang & Zhang, 2022; Y. Zhang et al., 2017). Empirical studies have also shown that children who adhere to 24 h movement guidelines demonstrate better cognitive performance (Cai et al., 2025; Yang et al., 2024). Regarding cognitive function, previous research has examined the relationship between 24 h movement behaviors and performance IQ in school-aged children, finding that the proportion of time spent in MVPA was significantly and positively associated with the total performance IQ score, whereas the proportion of time spent in SED was significantly and negatively associated with this score. Isotemporal substitution analysis further revealed that reallocating just 15 min between behaviors could produce significant changes in the predicted total performance IQ score (L. Guo et al., 2025). Regarding executive function, a study of children aged 4–6 years showed that isotemporal substitution of MVPA for SED or LPA could significantly improve executive function, whereas the reverse substitution led to a rapid decline (Chang et al., 2025). In addition, a systematic review encompassing 81,857 children and adolescents explicitly indicated that physical activity and a sedentary lifestyle were significantly associated with cognitive function in children and adolescents (D. Zhang et al., 2019). However, most of these studies focused their measurement of cognitive function on dimensions such as performance IQ and executive function, without addressing creative thinking, a more complex and higher-order cognitive construct.

Creative thinking is related to general cognitive ability but also differs from it in fundamental ways. Creative thinking emphasizes novelty, originality, and unconventional approaches to problem solving, and its cognitive mechanisms involve multiple dimensions, including divergent thinking, associative thinking, cognitive flexibility, and insight (Li et al., 2023). These dimensions differ substantially from executive function and fluid intelligence in their requirements for the behavioral environment. From a theoretical perspective, MVPA may influence creative thinking through neurophysiological mechanisms such as increasing cerebral blood flow, promoting the secretion of brain-derived neurotrophic factor, and improving prefrontal cortex function (Lu & Deng, 2021); SLP, particularly rapid eye movement sleep, plays a key role in memory reorganization and remote associations (Z. Wang & Hu, 2021); and excessive SED may inhibit the emergence of creative thinking by reducing cognitive arousal and limiting opportunities for environmental exploration (K. Zhang & Du, 2024; Bai & Yao, 2018). However, these theoretical propositions have yet to be systematically tested from a holistic perspective on 24 h movement behaviors. More importantly, the 24 h in a day constitutes a fixed total, and an increase in time spent in one movement behavior necessarily comes at the expense of time spent in other movement behaviors. Examining the relationship between a single movement behavior and creative thinking in isolation, as in traditional research, not only fails to reveal compensation and competition between different movement behaviors but may also produce statistical bias by ignoring the compositional nature of movement behaviors as a whole. The introduction of compositional data analysis (CoDA) and isotemporal substitution models provides a methodological breakthrough for accurately estimating the effects of time reallocation between different movement behaviors while maintaining a constant total of 24 h.

However, it remains unclear whether the composition of 24 h movement behaviors is associated with creative thinking in school-aged children, and whether reallocating time among SLP, SED, LPA, and MVPA is associated with changes in creative thinking. To our knowledge, no study has applied compositional data analysis and isotemporal substitution models to examine this question in this population. This study therefore addresses this gap by examining the associations between the composition of 24 h movement behaviors and creative thinking in school-aged children. This study focuses on the following issues: (1) the distribution of time spent in 24 h movement behaviors among school-aged children and its overall association with creative thinking; (2) the relationship between isotemporal substitution and creative thinking among the components of 24 h movement behaviors in school-aged children within a CoDA framework; and (3) the model-estimated reallocation curves relationship between progressively longer durations of substitution among MVPA, LPA, SED, and SLP and creative thinking in school-aged children. This study aims to provide a scientific basis for cultivating children’s creative thinking through the optimization of daily behaviors, while extending the boundaries of cognitive validity in research on 24 h movement behaviors and advancing this field from general cognitive function toward higher-order cognitive processes.

## 2. Materials and Methods

This was a cross-sectional observational study. The reporting of this study follows the Strengthening the Reporting of Observational Studies in Epidemiology (STROBE) guidelines for cross-sectional studies.

### 2.1. Participants

**Figure 1:**
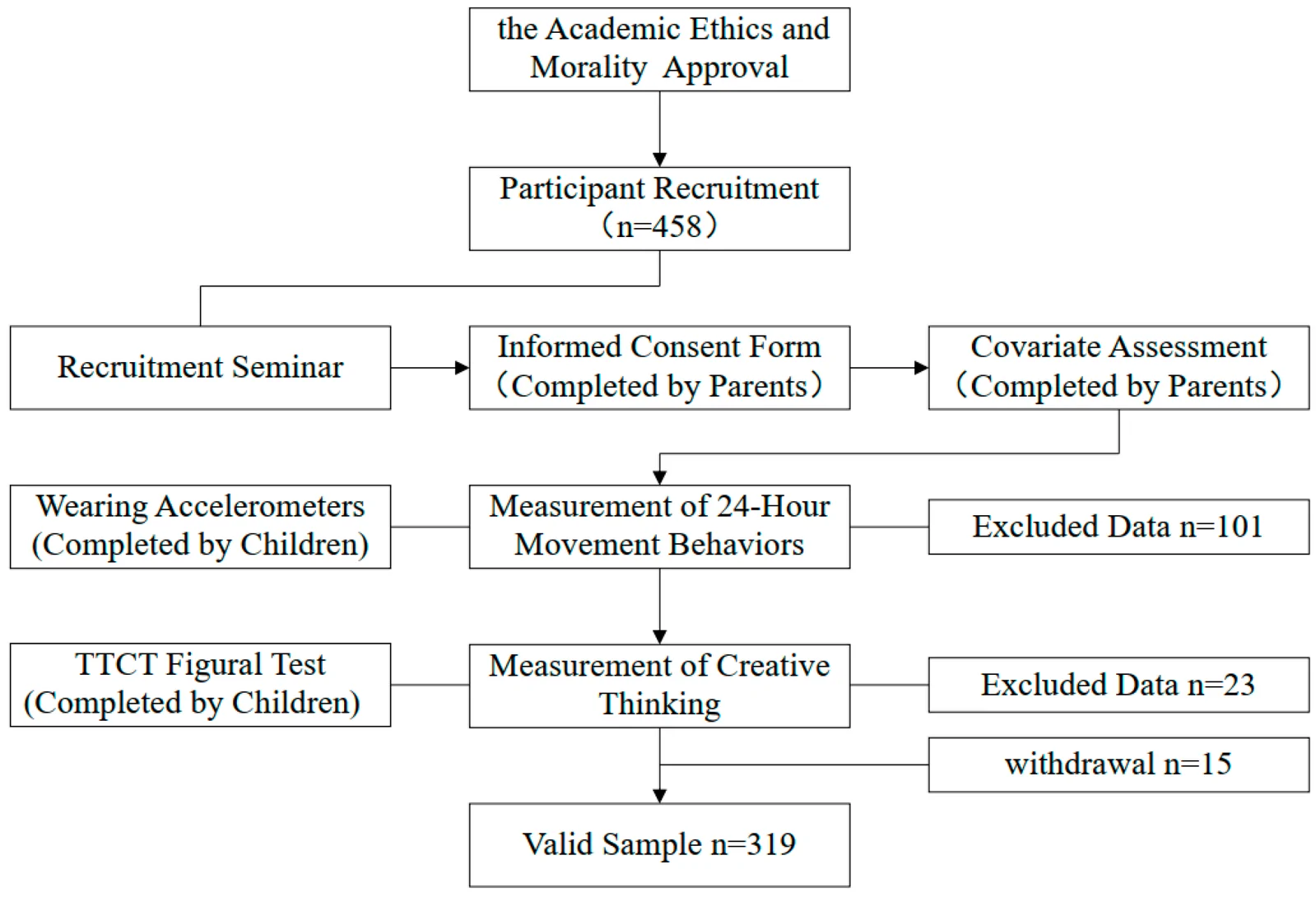
The data collection process.

Three public primary schools in Xuhui District, Shanghai, were selected using convenience sampling. Because of this sampling strategy, the findings are generalizable only to comparable urban Chinese primary-school populations, and not to all school-aged children. Eligible participants were Grade 1–5 students aged 6–12 years who: (1) had no diagnosed physical, neurological, or psychiatric condition that would preclude accelerometer wear or cognitive assessment; (2) provided written informed consent from a parent or guardian and oral assent; and (3) completed the accelerometer and TTCT assessments. The exclusion criteria were: (1) invalid 24 h movement behavior data (*n* = 101); (2) non-participation in or invalid TTCT data (n = 23); and (3) withdrawal during the assessments (n = 15). Of the 458 invited students, 319 were included in the final analysis. The data collection process is shown in Figure 1. The final sample comprised 319 children (168 boys, 151 girls; mean age = 10.06 ± 1.37 years; Grades 1–5: n = 65, 71, 59, 61, and 63, respectively). The study was approved by the Academic Ethics and Morality Committee of Shanghai Normal University (Approval No. SHNU Ethics [2024] No. 167, 19 November 2024).

No a priori sample size calculation was performed because this study used an existing cross-sectional dataset. The achieved sample size (n = 319) provided acceptable precision for the main compositional coefficients, as reflected in the 95% CIs reported in Table. We did not perform retrospective power calculations.

### 2.2. Measurement of 24 h Movement Behaviors

An ActiGraph GT3-BTX (ActiGraph LLC, Pensacola, FL, USA) activity and energy expenditure monitor (hereafter referred to as an accelerometer) was used to measure students’ 24 h movement behaviors over one week. Before the assessment, the sampling interval was set to 15 s, and the purpose of the assessment and relevant precautions were explained to the participants. During the assessment, participants were required to wear the accelerometer on the right hip for seven consecutive days, except during water-based activities. After the assessment, the data were processed using ActiLife (v 6.11.4) Valid assessment data were obtained according to the screening criteria for valid physical activity data proposed by Anderson et al. (2005), namely, at least 10 h of wear time per day and at least three valid days of wear within one week. The physical activity intensity classification criteria proposed by Zhu et al. (2013) for Chinese school-aged children were applied, using cut-points of 0, 100, 2800, and 4000 counts/min to classify movement behaviors as SED, LPA, moderate physical activity (MPA), and vigorous physical activity (VPA). Total daily SLP duration was calculated using the sleep algorithm proposed by Barreira et al. (2015). Participants’ daily time spent in MVPA (MPA + VPA), LPA, SED, and SLP was analyzed.

### 2.3. Measurement of Creative Thinking

The Torrance Tests of Creative Thinking (TTCT), developed by the American scholar Torrance (1984) and revised by the Chinese scholars Ye et al. (1988), were used. Given the limitations of children’s reading ability, the figural tests of the TTCT were selected to assess children’s creative thinking, including Picture Construction, Picture Completion, and Parallel Lines. The dimensions assessed were fluency, originality, elaboration, abstractness of titles, and resistance to premature closure. A higher total score across the five dimensions indicated stronger creative thinking. Scoring was based primarily on the Evaluation Manual. The assessment of fluency was entirely objective, whereas the other four dimensions were assessed using a ‘triangulation method’: three graduate students majoring in psychology scored the responses, and their mean score was used as the final score. The TTCT figural test was administered in a quiet classroom in small groups of 20–30 students by trained research assistants using standardized instructions. Each of the three activities lasted 10 min, and the total administration time was approximately 30 min. The TTCT was administered after the 7-day accelerometer monitoring period, within the same semester. The assessment setting was kept quiet and free from interruption. In this study, Cronbach’s α for the test was 0.744. The correlations among the five dimensions ranged from 0.213 to 0.406 and were all statistically significant; they were also lower than the correlations between each dimension and the total score, which ranged from 0.507 to 0.811. These findings indicated that the assessment method used in this study had high reliability and validity.

### 2.4. Covariates

Existing research has shown that sex, age, parental educational attainment, and family economic circumstances can influence students’ 24 h movement behaviors (Yang et al., 2023; Chen et al., 2021). Therefore, a questionnaire was used to collect data on participants’ sex, age, parental educational attainment (primary/junior secondary school, senior secondary/technical secondary school, college/university, and a master’s degree or above), and monthly household income (RMB 0–2000, RMB 2001–5000, RMB 5001–10,000, and ≥RMB 10,001). The questionnaire was completed by parents or guardians. Trained research assistants distributed the questionnaire during the same week as accelerometer monitoring, provided standardized instructions, and collected it upon completion. The questionnaire took approximately 10–15 min to complete. Missing covariate data were inspected before analysis; cases with missing covariates were excluded listwise from the main models.

### 2.5. Statistical Analysis

Data analysis followed the guidelines for CoDA of 24 h movement behaviors proposed by Chastin et al. (2015) and was performed using R (v 4.3.0) and Stata (v 17.0). The specific analytical steps were as follows:

(1) Analysis of the central tendency and dispersion of children’s 24 h movement behavior durations. Using descriptive statistical methods for compositional data (Chastin et al., 2015), compositional geometric means were used to describe the central tendency of children’s 24 h movement behavior data, and a variation matrix was used to describe the dispersion of the compositional data for SED, SLP, LPA, and MVPA. Smaller variance values indicated stronger relative codependence between two behaviors, whereas larger variance values indicated weaker relative codependence.
(2) Compositional regression analysis of the proportions of time spent in children’s 24 h movement behaviors. Based on the isometric log-ratio transformation used in compositional statistics (Liang et al., 2022; Chang & Wang, 2020), a regression model was constructed with SED, SLP, LPA, and MVPA as independent variables and the total creative thinking score as the dependent variable to examine the association between the proportions of time spent in children’s 24 h movement behaviors and creative thinking. Age, sex, parental educational attainment, and monthly household income were included in the regression model as control variables.
(3) Analysis of the compositional substitution effects of children’s 24 h movement behaviors. A ‘one-to-one’ isotemporal substitution approach was used to analyze the relationship between substitution among children’s 24 h movement behaviors and creative thinking. Following previous studies of compositional isotemporal substitution analysis (Dumuid et al., 2018, 2019), 15 min was first reallocated from one behavior to another while keeping the total time (1440 min) and time spent in the other behaviors constant. The difference in creative thinking scores between the reallocated time distribution and the original mean time distribution was then calculated (Dumuid et al., 2019; Haszard et al., 2020).
(4) Model-estimated reallocation curves analysis of children’s 24 h movement behaviors and creative thinking. For the pathways showing significant substitution effects in the substitution analysis, the reallocated duration was progressively increased in 5 min increments (Guan & Zhu, 2013; Dumuid et al., 2019) up to 60 min to further examine the model-estimated reallocation curves relationship between different substitution durations and the total creative thinking score.

## 3. Results

### 3.1. Participant Characteristics

Detailed participant characteristics are presented in Table 1. Parental education and household income distributions are also shown in Table 1. Among the categories of parental educational attainment, college/university education accounted for the largest proportion (43.0%), and primary/junior secondary school education accounted for the smallest proportion (5.6%); senior secondary/technical secondary school education accounted for 30.1%, and a master’s degree or above accounted for 21.3%. Among the categories of monthly household income, RMB 5001–10,000 accounted for the largest proportion (51.4%), and RMB 0–2000 accounted for the smallest proportion (4.1%); RMB 2001–5000 accounted for 25.1%, and ≥RMB 10,001 accounted for 19.4%. The mean creative thinking score was 63.32 ± 8.07.

### 3.2. Distribution of Participants’ Time Spent in 24 h Movement Behaviors

As shown in Table 2, compositional geometric means were used to describe the central tendency of participants’ time-use data for 24 h movement behaviors. The compositional geometric means for MVPA, LPA, SED, and SLP were 38.52 min (2.68%), 230.14 min (15.98%), 561.72 min (39.01%), and 609.62 min (42.33%), respectively, whereas the corresponding arithmetic means were 40.07 min (2.78%), 233.19 min (16.19%), 558.63 min (38.79%), and 608.11 min (42.23%). These results indicated some differences between the two types of means in their descriptions of central tendency. Compared with the compositional geometric means, the arithmetic means overestimated time spent in MVPA and LPA to some extent and underestimated time spent in SED and SLP to some extent.

As shown in Table 3, a variation matrix was used to describe the dispersion among the components of participants’ time-use data for 24 h movement behaviors. All pairwise log-ratio variances were greater than zero, indicating that the ratios between behaviors varied across participants. The smallest pairwise log-ratio variance was observed between SED and SLP (0.05), indicating the strongest relative codependence, whereas the largest was observed between SLP and MVPA (0.16), indicating the weakest relative codependence.

### 3.3. Compositional Linear Regression Analysis of Participants’ 24 h Movement Behaviors and Creative Thinking

As shown in Table 4, after adjustment for covariates, compositional regression analysis was performed with the components of 24 h movement behaviors as the independent variables and creative thinking as the dependent variable to examine the association between participants’ time distribution of 24 h movement behaviors and creative thinking. The proportions of time spent in MVPA (β = 16.019, 95%CI [9.472, 22.578], *p* < 0.001) and SLP (β = 6.374, 95%CI [3.263, 9.492], *p* < 0.001) were significantly and positively associated with creative thinking. Specifically, total creative thinking scores increased as time spent in MVPA increased (with relative decreases in LPA, SED, and SLP) or as time spent in SLP increased (with relative decreases in MVPA, LPA, and SED). The proportion of time spent in SED (β = −11.621, 95%CI [−19.345, −3.906], *p* < 0.01) was significantly and negatively associated with total creative thinking scores; that is, total creative thinking scores decreased as time spent in SED increased (with relative decreases in MVPA, LPA, and SLP). The association between the proportion of time spent in LPA (β = −4.284, 95%CI [−8.771, 0.207], *p* = 0.062) and total creative thinking scores was negative but not statistically significant.

### 3.4. Substitution Among Participants’ 24 h Movement Behaviors and Changes in Total Creative Thinking Scores

As shown in Table 5, a 15 min substitution duration was used to examine the effects of mutual substitutions among MVPA, LPA, SED, and SLP on creative thinking. The results showed that: (1) replacing 15 min of LPA or SED with MVPA increased predicted creative thinking scores by 1.887 and 2.265 points, respectively; conversely, replacing 15 min of MVPA with LPA or SED decreased predicted creative thinking scores by 2.375 and 2.636 points, respectively. (2) Replacing 15 min of LPA or SED with SLP increased predicted creative thinking scores by 0.681 and 1.207 points, respectively; conversely, replacing 15 min of SLP with LPA or SED decreased predicted creative thinking scores by 0.994 and 1.311 points, respectively. (3) Mutual 15 min substitutions between MVPA and SLP, or between LPA and SED, produced no significant changes in creative thinking.

With a standard deviation of 8.07 for the TTCT total score, the 15 min substitution estimates corresponded to approximately 0.08–0.33 SD. For example, replacing 15 min of LPA with MVPA was associated with a 1.887-point higher predicted score, corresponding to 0.23 SD; replacing 15 min of SED with MVPA was associated with a 2.265-point higher predicted score, corresponding to 0.28 SD. The reverse substitutions corresponded to approximately −0.29 to −0.33 SD. These are small-to-moderate model-estimated differences, and they may be relevant at the population level if sustained.

### 3.5. Model-Estimated Reallocation Curves Analysis of Substitution Among Participants’ 24 h Movement Behaviors and Creative Thinking

**Figure 2:**
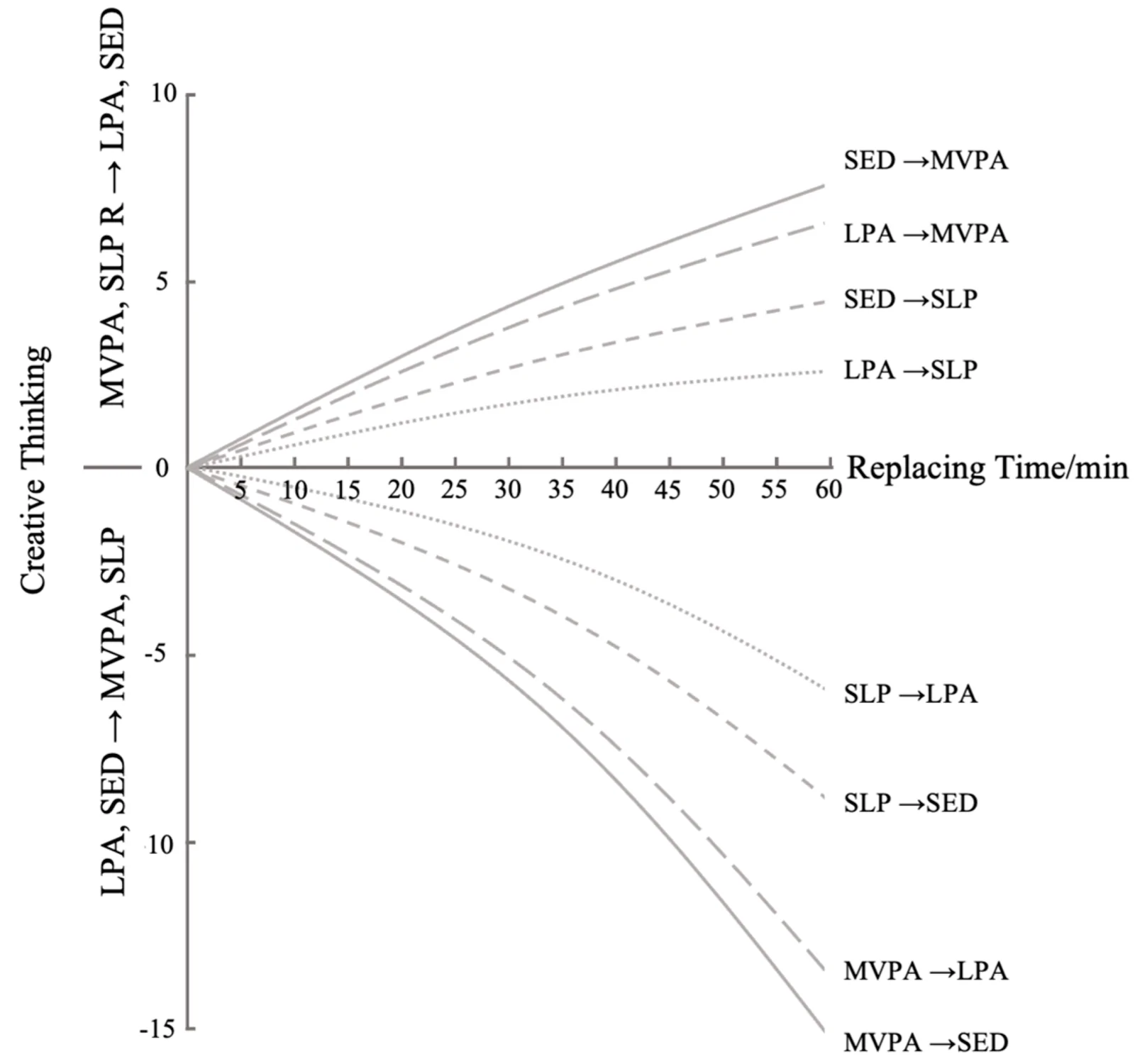
Changes in creative thinking following isotemporal substitution of MVPA or SLP for LPA or SED.

As shown in Figure 2, for the pathways with significant substitution effects in the substitution analysis, the substitution duration was further increased in 5 min increments up to 60 min to examine the model-estimated reallocation curves’ relationship between substitution duration and creative thinking. The results showed that: (1) the model-estimated reallocation curves' relationships between mutual substitutions between MVPA and LPA or SED and creative thinking were markedly asymmetric. Specifically, creative thinking increased gradually as more time spent in LPA or SED was replaced by MVPA, whereas it decreased rapidly when the substitutions occurred in the opposite direction. The model-estimated reallocation curves show that relationships between mutual substitutions between SLP and LPA or SED and creative thinking were also markedly asymmetric: creative thinking increased gradually as more time spent in LPA or SED was replaced by SLP, whereas it also decreased rapidly when the substitutions occurred in the opposite direction. (2) Creative thinking showed a continuous upward trend as more time spent in LPA or SED was replaced by MVPA or SLP. For both MVPA and SLP, the increase was greater when replacing SED than when replacing LPA.

## 4. Discussion

This study introduced compositional data analysis (CoDA) into research on physical activity and creative thinking to systematically examine the relationship between 24 h movement behaviors and creative thinking in school-aged children in China, addressing collinearity in conventional statistical analyses and comprehensively revealing the overall associations between 24 h movement behaviors and creative thinking in this population. The results showed that the proportions of time spent in MVPA and SLP were significantly and positively associated with creative thinking in school-aged children, whereas the proportion of time spent in SED was significantly and negatively associated with creative thinking; the association with the proportion of time spent in LPA was negative but not statistically significant (β = −4.284, *p* = 0.062). These cross-sectional findings suggest that relative time in MVPA and SLP is associated with higher creative thinking scores in school-aged children and suggest that MVPA and SLP may positively influence its development. If these associations are causal, increasing MVPA and SLP while reducing SED and LPA could be a promising pathway; however, intervention studies are needed to test this possibility. This is generally consistent with existing research findings (Feng et al., 2025; Lim et al., 2025; Li et al., 2023; Romance et al., 2023; Frith et al., 2019; Lewis et al., 2018). The study also yielded the following main findings:

(1) The variation matrix analysis indicated relative codependence among MVPA, LPA, SED, and SLP in school-aged children, with the strongest relative codependence between SED and SLP (log-ratio variance = 0.05). Time reallocations between SED and SLP may involve particularly study-related and screen-related sedentary time in primary school children. One possible explanation, which requires future testing, is that academic demands and parental expectations in some Chinese families may lead to trade-offs between sleep and academically oriented sedentary time (Sun et al., 2023; Man et al., 2016). Screen-related sedentary time may also be reallocated to or from sleep. As the Internet and electronic devices become increasingly widespread, primary school children may readily develop habits of sedentary screen use, although these behaviors are often subject to parental intervention and restrictions (P. Wang et al., 2023; Zhao et al., 2022).

In addition, this study found comparatively weak codependence between MVPA and the other movement behaviors in school-aged children, with the weakest relative codependence between MVPA and SLP (log-ratio variance = 0.16). These findings suggest that the associations of MVPA and SLP with creative thinking may be relatively independent. MVPA may influence creative thinking through neurophysiological mechanisms such as increasing cerebral blood flow, promoting BDNF secretion, and improving prefrontal cortex function (Lu & Deng, 2021); SLP, particularly REM sleep, may support memory reorganization and remote associations (Z. Wang & Hu, 2021); and excessive SED may inhibit creative thinking by reducing cognitive arousal and limiting environmental exploration (K. Zhang & Du, 2024; Bai & Yao, 2018). These mechanisms are theoretically plausible but were not measured in the present study. However, Maher et al. (2014) argued that the associations of MVPA, LPA, SED, and SLP with physical and mental health are not independent, because an increase in time spent in one or more movement behaviors necessarily results in a decrease in time spent in one or more other movement behaviors. Moreover, the CoDA approach used in this study also follows the fundamental principle that a change in time spent in any movement behavior necessarily entails changes in the time allocated to the remaining movement behaviors.

(2) The compositional isotemporal substitution models estimated significant associations of bidirectional substitutions between MVPA or SLP and LPA or SED on the development of creative thinking in school-aged children. Replacing an equal duration of LPA or SED with an average of 15 min of MVPA or 15 min of SLP per day was associated with significant improvements in creative thinking in school-aged children. These findings suggest that increasing time spent in MVPA and SLP plays an important role in improving creative thinking in school-aged children, further supporting previous findings that necessary MVPA and adequate SLP are most beneficial to the healthy development of children and adolescents (Xing et al., 2025; Yang et al., 2024; Gao et al., 2024). This is also consistent with existing physical activity guidelines for school-aged children and the principles of sleep management for primary and secondary school students. These guidelines advocate increasing physical activity levels and reducing sedentary time in school-aged children while emphasizing adequate sleep, indicating that reducing SED and ensuring sufficient SLP and MVPA are important for the physical and mental health development of school-aged children (Ministry of Education of the People’s Republic of China, 2021; H. Guo & Rao, 2021; Y. Zhang et al., 2017).

The isotemporal substitution analysis also showed that, when MVPA or SLP replaced an equal duration of LPA or SED in school-aged children, the model-estimated differences for replacing SED were consistently greater than those of replacing LPA. Considered alongside these findings, the variation matrix analysis showed that SED had the strongest relative codependence with SLP and stronger relative codependence with LPA than with MVPA. The substitution results therefore suggest that reducing LPA and increasing MVPA may be an important pathway for promoting the development of creative thinking in school-aged children. The amount of time available in a day is limited, and SED cannot be reduced indefinitely. This reminds parents and educators to gradually reconsider the entrenched assumption that “less sedentary time = sufficient physical activity = good physical and mental health” and to help school-aged children appropriately increase the proportion of time allocated to MVPA during their available active time while ensuring an appropriate proportion of SLP, thereby better promoting their physical and mental health development (Chang & Wang, 2022; Chang, 2021; Chang & Wang, 2020).

(3) The model-estimated reallocation curves analysis revealed marked asymmetry in the effects of bidirectional substitutions between MVPA or SLP and LPA or SED in school-aged children: creative thinking scores increased gradually as MVPA or SLP replaced LPA or SED, but decreased rapidly when LPA or SED replaced MVPA or SLP. For example, replacing 15 min of LPA with 15 min of MVPA was associated with a 1.887-point increase in creative thinking, whereas the reverse substitution was associated with a 2.375-point decrease; these effects became more pronounced as the duration of isotemporal substitution increased. Similar asymmetry in substitution effects has been reported in previous compositional substitution studies of physical fitness and mental health (Yang et al., 2024; Chang & Wang, 2022; Liang et al., 2022; Chang & Wang, 2020). This may be related to the proportion of a behavior’s total duration represented by the reallocated time. In this study, theoretically, MVPA + LPA + SED + SLP = 1440 min; MVPA accounted for only 2.68% of the compositional mean, whereas SED and LPA accounted for 39.01% and 15.98%, respectively. Removing 15 min of MVPA would represent nearly one-third of the MVPA time (38.52 min) of the school-aged children in this study, and reallocating such a large proportion of time to other behaviors would naturally produce a larger substitution effect. Conversely, removing 15 min of SED would represent only 1/37 of the children’s SED time (561.72 min), so the effect of this small adjustment would also be small. Thus, a 15 min change represents a substantial change in MVPA but only a minor adjustment in SED, which may account for the asymmetry in the benefits of isotemporal substitution. Therefore, the steeper curves for MVPA-donor substitutions may partly reflect the mathematical properties of the composition rather than a stronger physiological effect of MVPA. This distinction is important: the asymmetry is a property of the model and the observed time-use distribution, and it should not be interpreted as direct evidence of different physiological dose–response mechanisms. In addition, previous studies of substitutions within 24 h movement behaviors in relation to children’s physical fitness have identified a “cumulative effect” of physical activity (Liang et al., 2022; Chang & Wang, 2020). Individuals seeking to improve their physical fitness need to maintain regular exercise to achieve greater fitness benefits; conversely, these benefits may decline rapidly when individuals fail to maintain or stop exercising. This “cumulative effect” may also apply to the influence of 24 h movement behaviors on creative thinking in school-aged children. The apparent asymmetry in substitution associations may reflect a cumulative effect of physical activity, but this mechanism cannot be established from cross-sectional data. The possibility of a cumulative physiological effect of physical activity remains a hypothesis requiring longitudinal or intervention testing.

This study has several strengths. To our knowledge, it is the first to apply compositional data analysis and compositional isotemporal substitution models to examine the association between 24 h movement behaviors and creative thinking in school-aged children. Movement behaviors were measured objectively using accelerometers over seven consecutive days. The relatively large sample, adjustment for key covariates, and dose–response analysis further strengthen the robustness of the findings. Several limitations warrant consideration. First, the cross-sectional design precludes causal inference, and the substitution associations are model-based estimates rather than experimentally verified effects; reverse causality cannot be excluded, as children with higher creative thinking may be more likely to engage in MVPA, maintain regular sleep, or spend less time sedentary. Second, convenience sampling from three primary schools in a single district of Shanghai, together with a final exclusion rate of approximately 30%, limits generalizability and raises the possibility of selection bias; students were also nested within schools and classes, and although clustering was not formally modeled because of the small number of higher-level units, future studies should employ multilevel models or cluster-robust standard errors. Third, residual confounding may persist, including diet, parental involvement, school environment, and baseline cognitive ability. Fourth, measurement limitations include accelerometer cut-points, non-wear time, the sleep algorithm, and valid-day criteria, all of which may introduce misclassification; sedentary behavior subtypes were not distinguished, and inter-rater uncertainty in the TTCT cannot be fully excluded. Finally, swimming and other water-based MVPA were not captured. This may underestimate MVPA and slightly distort the 24 h movement behavior composition, particularly for children who frequently swim. Future research should use longitudinal designs, cluster-randomized or compositional intervention trials, objective measures, posture-sensitive devices, and multidimensional creativity assessments. Studies should also examine whether reallocating time from SED and LPA to MVPA and SLP produces measurable changes in creative thinking.

## 5. Conclusions

In this cross-sectional compositional analysis, relative time in MVPA and SLP was positively associated with creative thinking in school-aged children, whereas relative time in SED was negatively associated. Model-estimated reallocations from LPA or SED to MVPA or SLP were associated with higher predicted creative thinking scores, and reverse reallocations were associated with lower predicted scores. These associations were asymmetric, and replacing SED yielded greater model-estimated differences than replacing LPA. These findings are cross-sectional and cannot establish causality. Future longitudinal and intervention studies are needed to test whether reallocating time among 24 h movement behaviors causally influences creative thinking. Pending such evidence, families, schools, and society may consider supporting adequate MVPA and sleep and reducing sedentary time as part of a holistic approach to child development; however, these suggestions should be regarded as potential practical implications requiring confirmation, not as evidence-based causal recommendations.

## Figures and Tables

**Table 1:** Detailed participant characteristics.

Statistic		n	%
Sex	boys	168	52.7
	girls	151	47.3
Grade	1	65	20.4
	2	71	22.3
	3	59	18.5
	4	61	19.1
	5	63	19.7
Parental education	primary/junior secondary	18	5.6
	senior secondary/technical secondary	96	30.1
	college/university	137	43.0
	master’s or above	68	21.3
Monthly household income (RMB)	0–2000	13	4.1
	2001–5000	80	25.1
	5001–10,000	164	51.4
	≥10,001	62	19.4

**Table 2:** Compositional and arithmetic means of participants’ time-use data for 24 h movement behaviors.

Statistic	SLP	SED	LPA	MVPA
Compositional mean, min	609.62	561.72	230.14	38.52
Compositional mean, %	42.33	39.01	15.98	2.68
Arithmetic mean, min	608.11	558.63	233.19	40.07
Arithmetic mean, %	42.23	38.79	16.19	2.78

**Table 3:** Variation matrix of participants’ time-use data for 24 h movement behaviors.

Movement Behavior	SLP	SED	LPA	MVPA
SLP	0.00	0.05	0.08	0.16
SED	0.05	0.00	0.12	0.14
LPA	0.08	0.12	0.00	0.15
MVPA	0.16	0.14	0.15	0.00

**Table 4:** Compositional linear regression of participants’ relative time spent in 24 h movement behaviors and total creative thinking scores.

Movement Behavior	β	95%CI	*p*	Model *p*	Model *R*²
SLP	6.374	[3.263, 9.492]	<0.001	0.001	0.393
SED	−11.621	[−19.345, −3.906]	0.004		
LPA	−4.284	[−8.771, 0.207]	0.062		
MVPA	16.019	[9.472, 22.578]	<0.001		

Note: β represents a model-estimated association. It reflects the change in TTCT total score associated with a change in one movement behavior relative to the remaining movement behaviors.

**Table 5:** Changes in total creative thinking scores following 15 min isotemporal substitutions among SLP, SED, LPA, and MVPA.

Donor Behavior (↓ 15 min)	Receiver: SLP ↑	Receiver: SED ↑	Receiver: LPA ↑	Receiver: MVPA ↑
SLP ↓		−1.311 [−2.426, −0.943] *	−0.994 [−1.483, −0.748] *	0.173 [−0.102, 0.336]
SED ↓	1.207 [0.674, 2.768] *		0.216 [−0.105, 0.427]	2.265 [1.083, 3.001] *
LPA ↓	0.681 [0.043, 1.849] *	−0.314 [−1.651, 0.966]		1.887 [0.947, 2.984] *
MVPA ↓	0.132 [−0.048, 0.292]	−2.636 [−3.539, −1.132] *	−2.375 [−3.877, −1.129] *	

Note: Values are predicted changes (95% confidence intervals [CI]); * indicates *p* < 0.05; ↑ indicates a 15 min increase in time spent in the specified movement behavior; ↓ indicates a 15 min decrease.

## Data Availability

The anonymized dataset and analysis code are available from the corresponding author upon reasonable request, subject to ethical approval.

## Abbreviations

CoDA	compositional data analysis
MVPA	moderate-to-vigorous physical activity
LPA	light physical activity
SED	sedentary behavior
SLP	sleep
TTCT	Torrance Tests of Creative Thinking
MPA	moderate physical activity
VPA	vigorous physical activity
CI	confidence interval
SD	standard deviation
IRB	institutional review board.
